# Bacterial contamination of chicken meat in slaughterhouses and the associated risk factors: A nationwide study in Thailand

**DOI:** 10.1371/journal.pone.0269416

**Published:** 2022-06-08

**Authors:** Kunnanut Klaharn, Duangporn Pichpol, Tongkorn Meeyam, Thanida Harintharanon, Patpong Lohaanukul, Veerasak Punyapornwithaya

**Affiliations:** 1 Faculty of Veterinary Medicine, Department of Veterinary Bioscience, Chiang Mai University, Chiang Mai, Thailand; 2 Faculty of Veterinary Medicine, Veterinary Public Health Research Group, Chiang Mai University, Chiang Mai, Thailand; 3 Faculty of Veterinary Medicine, Veterinary Public Health and Food Safety Centre for Asia Pacific (VPHCAP), Chiang Mai University, Chiang Mai, Thailand; 4 Department of Livestock Development, Bangkok, Thailand; University of Bologna, ITALY

## Abstract

Slaughterhouses are a key source of bacterial contamination in poultry meat and products, which is a major health and economic concern for several public authorities. This study aimed to quantify the non-compliance of bacterial contamination on chicken meat sampled from slaughterhouses and identify risk factors associated with the contamination. A questionnaire survey of 569 chicken slaughterhouses was undertaken and 1,707 meat samples were collected to determine the level of bacterial contamination. The proportion of the non-compliance associated with aerobic plate count [APC] (24.6%), *Staphylococcus aureus* (6.3%), *Enterococcus* spp. (24.7%), coliforms (13.5%), *Escherichia coli* (33.3%), and *Salmonella* spp. (33.4%) based on the livestock authorities’ criteria was determined. Our results highlighted that the scalding process without scalding water temperature control or improper scalding increased the risk of APC (odds ratio, OR = 4.84, 95% CI: 2.72–8.61), *S*. *aureus* (OR = 2.68, 95% CI: 1.29–5.55), *Enterococcus* spp. (OR = 3.38, 95% CI: 2.01–5.69), coliforms (OR = 3.01, 95% CI: 1.47–6.15), and *E*. *coli* (OR = 2.69, 95% CI: 1.58–4.56) contamination on meat samples. Meat from eviscerated carcasses was more likely to be non-compliance due to contamination by *E*. *coli* (OR = 1.96, 95% CI: 1.14–3.38). Furthermore, open or semi-closed system slaughterhouses (OR = 1.79, 95% CI: 1.23–2.60) and lack of equipment for specific slaughtering areas (OR = 1.65, 95% CI: 1.04–2.61) increased the likelihood of *Salmonella* spp. occurrence. This is the first study of factors influencing the non-compliance of meat samples across Thailand. Authorities can use the study findings to enhance food safety strategies at the national level.

## Introduction

Poultry meat production and consumption have substantially increased worldwide and are expected to rise in upcoming decades [1,2]. Interestingly, poultry meat consumption is the largest meat sector and has approximately doubled from 67 million tons in 2000 to 131 million tons in 2020 [3]. The key factors that make poultry meat preferable are relative affordability and economic costs in comparison to other meats, absence of religious or cultural restrictions, and dietary as well as nutritional properties [4].

Poultry meat is considered a potential vehicle for foodborne pathogens making it a major public health concern worldwide [5,6]. This has a high global impact on human health and socioeconomic burden [7–9]. Thailand reported 87,093 food poisoning cases with one death in the year 2020 [10]. Besides foodborne diseases, spoilage bacteria may decrease the shelf life leading to product losses in the poultry meat production industry causing substantial economic repercussions [11].

The bacterial contamination has been demonstrated to occur along the production chain from farm to fork including primary production at farm level along with live-poultry transportation, slaughtering processes, slaughterhouse environment, and in storage until it reaches consumer [12,13]. Slaughtering processes in slaughterhouses play an important role in foodborne microbial transmission. Contamination has been revealed to occur mostly during the slaughtering processes [14,15], with plucking, evisceration, and chilling being the most crucial processing steps [16–18]. Therefore, an improvement in hygienic practices across the food chain is necessary to reduce the risk of the foodborne burden from poultry meat products.

In Thailand, the Department of Livestock Development (DLD) implements a monitoring program to monitor levels of aerobic plate count (APC), *Staphylococcus aureus*, *Enterococcus* spp., coliforms, *Escherichia coli*, and *Salmonella* spp. in meat at slaughterhouses [19]. According to the monitoring program, despite evidence of high levels of bacterial contamination in meat samples [20], studies into potential causes of bacterial contamination during the slaughtering process in Thailand are very limited. Furthermore, because none of the studies was conducted on a national level, there is a lack of national baseline data, which is essential for formulating a national strategic plan to improve chicken meat quality across the country. Thus, this study aimed to determine the bacterial contamination status of the meat sampled from slaughterhouses and to identify the potential risk factors associated with non-compliance due to such contamination under the nationwide government-mandated quality control program.

## Material and methods

### Study area

**Fig 1:**
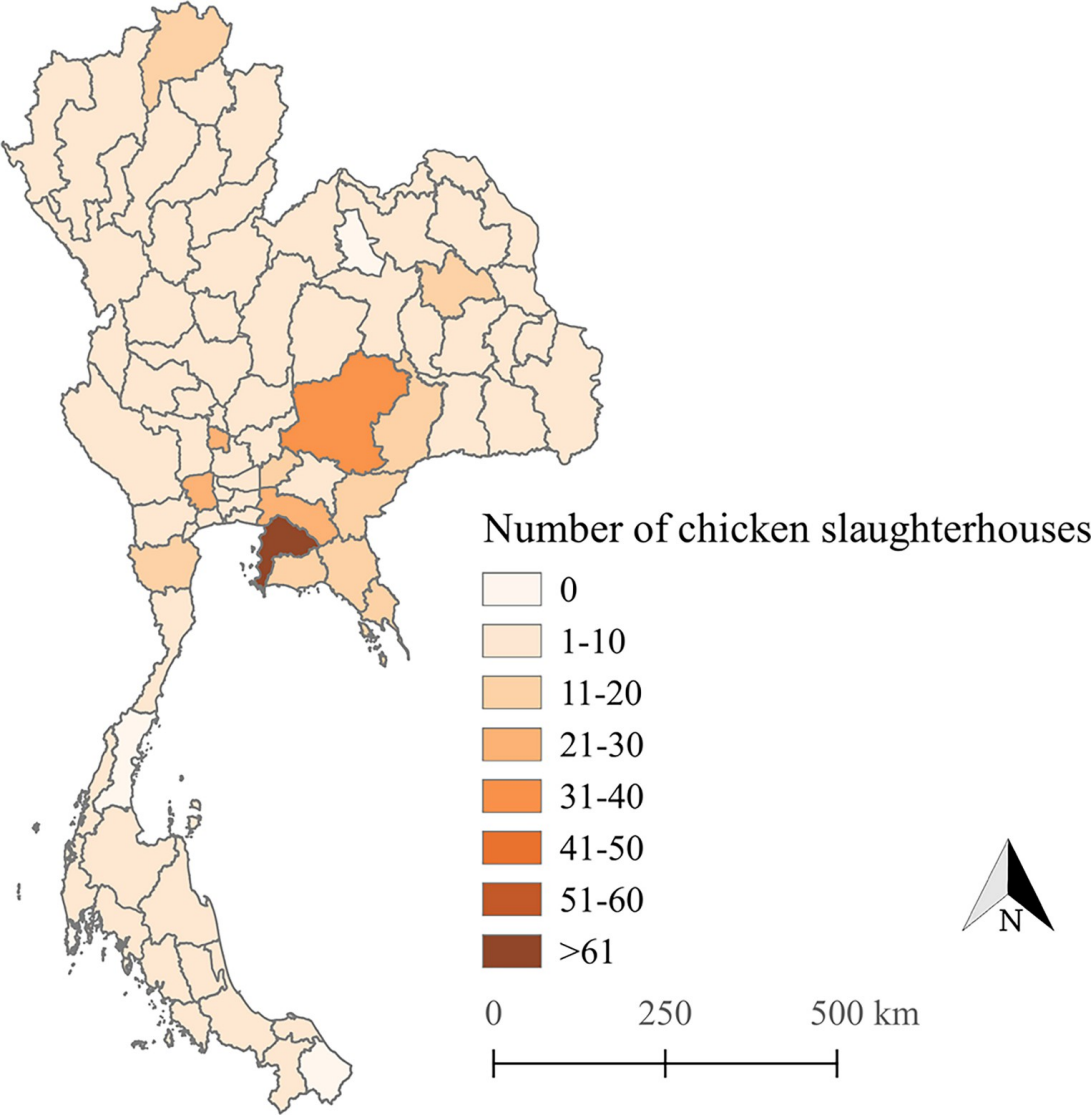
Map of Thailand showing the number of certified chicken slaughterhouses in each province. The map shown in Fig 1 was created using QGIS (version 2.18.28), QGIS Geographic Information System, and Open-Source Geospatial Foundation Project, and all content is licensed under Creative Commons Attribution-Share Alike 3.0 license (CC BY-SA), available at (http://qgis.osgeo.org). In addition, geographical materials used for creating the map (e.g., shape file) were supported by Chiang Mai University.

The study was conducted in 77 provinces across Thailand during 2019 and 2020 in chicken slaughterhouses (n = 569) under the authority of the DLD. Slaughterhouses that produce meat only for domestic consumption were included in this study. The number of chicken slaughterhouses in each province is depicted in Fig 1.

### Sample size calculation

The government program aimed to include all certified slaughterhouses in Thailand (n = 569), thus the number of slaughterhouses was influenced by this objective. Therefore, the sample size calculation was mainly considered on how many meat samples should be taken from each slaughterhouse. To perform this calculation, we used the online Power Analysis application designed to determine the sample size for a multilevel logistic regression analysis that corresponds to the study with a multilevel structure [21].

We calculated the power of the test using the following parameters: the number of meat samples taken from each slaughterhouse (the first level; n = 3), the number of slaughterhouses (the second level, n = 569), and the number of fixed-effect factors (n = 3). Since our factors were categorical variables, we specified the binomial distribution for them. Computer simulations (n = 100 times) were performed to estimate the power of the test for multilevel logistic regression. The results from the simulation showed that the statistical power was greater than 80%, which is generally accepted for statistical analysis [22].

### Meat sampling, microbiological analysis, and questionnaire survey

#### Meat sampling

Trained livestock authorities obtained three meat samples from each chicken slaughterhouse and submitted them (n = 1,707) to the DLD laboratory centers. Microbiological analysis was facilitated by the Bureau of Quality Control of Livestock Products (BQCLP) and Regional Veterinary Research and Development Centers. Meat samples from slaughterhouses were taken in accordance with the national monitoring program. The detail of meat sampling was previously described [23,24]. Briefly, each meat sample was randomly selected from an individual carcass by the authorities The samples were kept in an icebox to keep the temperature below 4°C. According to the DLD official guidelines, all samples were transported to DLD laboratory centers for microbiological analysis [23].

#### Microbiological analysis and regulatory criteria

The meat samples were analyzed according to the procedure given in FDA BAM *Online*, 2001 (Chapter 3) for aerobic plate count (APC); ISO 6888–1: 1999 for *Staphylococcus aureus*; Nordic Committee on Food Analysis, No. 68 5^th^ Edition (2011) for *Enterococcus* spp.; FDA BAM *Online*, 2013 (Chapter 4) for coliforms and *Escherichia coli*; and ISO 6579–1: 2017 for *Salmonella* spp. [25] (S1 Table). Microbiological criteria and the procedure used in this study were derived from the manual of Microbiological Standard for Livestock Products [26]. The criteria for determining whether meat samples comply with the standard value [27] are provided in S1 Table.

#### Questionnaire survey

Provincial livestock authorities visited all slaughterhouses during 2019–2020 and interviewed the slaughterhouse managers using a structured questionnaire to obtain data concerning slaughterhouse practices and facilities. Face-to-face interviews involving authorities and slaughterhouse supervisors have been used to collect data for the questionnaire. All data were reported to the Bureau of Livestock Standards and Certification (BLSC), which is officially in charge of monitoring livestock products at slaughterhouses across Thailand.

### Statistical analyses

#### Data and descriptive statistics

The outcome variable was the compliant status of the meat samples based on the DLD standard criteria (S1 Table) and was defined as a dichotomous variable. Factors derived from the questionnaire used for further analysis are listed in S2 Table.

Based on the DLD standard criteria, the contamination status of the meat sample was classified as either compliance or non-compliance (0 = compliance and 1 = non-compliance). The non-compliance of the meat samples for APC, *S*. *aureus*, *Enterococcus* spp., coliforms, *E*. *coli*, and *Salmonella* spp. contamination levels was evaluated. The proportion of the non-compliance and its 95% confidence interval (95% CI) was calculated based on Wald estimation [28].

#### Logistic regression analysis

Given the data structure, a multilevel mixed-effects logistic regression model was used to determine the association between slaughterhouse process (factor) and non-compliance of meat samples for each contamination criteria using R statistical software version 3.6.2 [29] with the “lme4” [30] and “dplyr” [31]. The analysis consisted of univariable and multivariable mixed-effects logistic regression analysis. For the univariable analysis, the association between each factor and the non-compliance was tested and factors with *p* < 0.2 from the univariable analysis were selected for the multivariable analysis.

The mixed-effect logistic regression accounted for the multilevel structure [32] is written as follow:

$$ln(\frac{P_{i}}{1-P_{i}})=\beta_{0}+\beta_{1}X_{1}+\cdots +\beta_{k}X_{k}+u_{j(i)}$$

where *P_i_* is the probability of non-compliant status of the *i^th^* meat sample, *β*_0_ is the model intercept, *X_k_* is the set of fixed-effects factors, and *β_k_* is the estimated coefficient according to the factors, *u*_*j*(*i*)_ is the random-effect of the *j^th^* slaughterhouse containing the *i^th^* meat sample and it was assumed that $u_{j(i)}∼NID(0,\sigma_{u}^{2})$.

Through model selection processes, a backward elimination technique was employed to determine the final model. Also, multicollinearity among factors was evaluated during the model selection process by examining variance inflation factors. Additionally, the logistic regression assumption of a linear relationship between factors and the logit of the outcome was evaluated by examining the scatter plot between each factor and the logit values. Moreover, a goodness of fit for each final model was tested using the Hosmer-Lemeshow test [33]. For each factor, the odds ratio and its 95% CI were calculated. The level of statistical significance was set as α = 0.05.

### Ethical statement

This study did not require ethical approval for animal research as live animals were not involved. As authorized staff from government sectors were involved in meat sampling, microbiological analysis and interview as part of the national survey organized by DLD, Thailand; no additional permission was required.

## Results

### Slaughterhouse characteristics

The characteristics of the slaughterhouses are shown in Table 1. In terms of slaughterhouse structure, nearly half of all slaughterhouses were closed-system buildings. Approximately 91.4% of slaughterhouses had separated slaughtering lines for clean and unclean areas. About 80% of the slaughterhouses have dedicated workers for a specific area and these workers do not have rotational duties at different areas within slaughterhouses. Likewise, the equipment is also not mixed within the specific areas and is strictly kept designated to a particular area. Before entering the slaughtering line, 77% of workers washed their hands and 76.5% wore protective clothing and boots. In less than half of the slaughterhouses, the slaughtering knife and cutting knife were sanitized and scalding water temperature in the scalding process was controlled. Only 20.9% of them had hanging equipment to prevent cross-contamination between chicken carcasses and the slaughtering floor. Even though approximately 50% of slaughterhouses did not use tap water, only 21.4% of slaughterhouses had water treatment systems in place prior to using water for the slaughtering processes. According to product characteristics, 68% of chicken carcasses processed in slaughterhouses were eviscerated.

**Table 1:** The characteristics of the chicken slaughterhouses based on the questionnaire survey.

Characteristics	n (N = 569)	%
**Slaughterhouse management practices**		
Water source; use of tap water in slaughterhouses	271	47.63
Slaughterhouses have the system to treat water used during slaughtering process	122	21.44
Separating workers for clean and unclean area	428	75.22
Separating equipment for clean and unclean area	458	80.49
Hand cleaning practice before entering the slaughtering area	438	76.98
Changing protective clothes and boots before entering the slaughtering area	435	76.45
Use of hanging equipment to prevent carcasses contamination	119	20.91
Slaughtering knives are sanitized before use	277	48.68
Cutting knives are sanitized before use	271	47.63
Having temperature control for scalding water	233	40.95
**Slaughterhouse designated and facilities**		
Close-system building	250	43.94
Separating slaughtering line for clean and unclean area	520	91.39
**Product characteristics**		
Eviscerated carcasses as the final products	387	68.01

### Bacterial contamination in chicken meat from slaughterhouses

**Fig 2:**
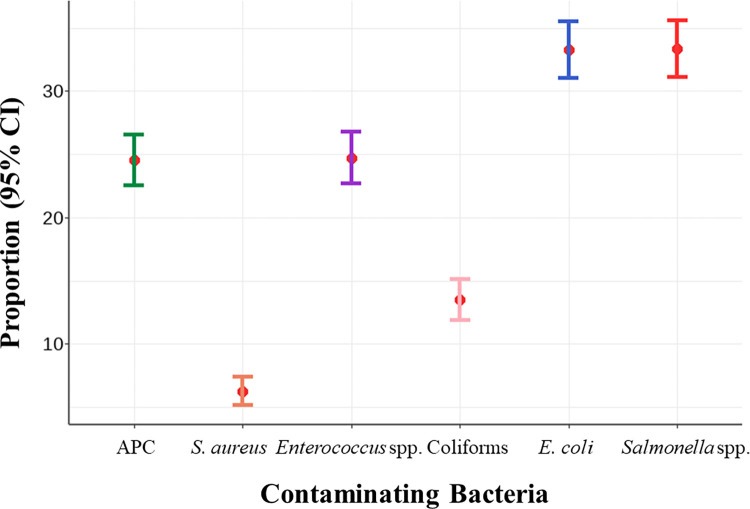
Proportion and 95% confidence interval (95% CI) of non-compliant meat samples according to the Department of Livestock Development criteria.

The proportion of non-compliant meat samples according to DLD criteria for aerobic plate count (APC), *Staphylococcus aureus*, *Enterococcus* spp., coliforms, *Escherichia coli*, and *Salmonella* spp. are shown in Fig 2. The results indicated *Salmonella* spp. contamination was the highest in the proportion of non-compliant meat samples (33.4%), followed by *E*. *coli* (33.3%) and *Enterococcus* spp. (24.7%), whereas the lowest proportion of non-compliant meat samples was *S*. *aureus* contamination (6.3%).

### Risk factors

The results from the multivariable logistic regression highlighted that improper scalding process increased the risk for APC (Table 2), *S*. *aureus* (Table 3), *Enterococcus* spp. (Table 4), coliforms (Table 5), and *E*. *coli* (Table 6) contamination in meat samples more than two-fold. Additionally, eviscerated carcasses were also associated with non-compliant meat samples for *E*. *coli*. Moreover, the factors associated with the non-compliant meat samples according to the contamination of *Salmonella* spp. are shown in Table 7.

**Table 2:** Factors associated with the aerobic plate count non-compliance of meat samples based on the univariable and multivariable mixed-effects logistic regression.

	Factors	Categories	Univariable analysis		Multivariable analysis	
			Odds ratio (95% CI)	*P*-Value	Adjusted OR*(95% CI)	*P*-Value
1	Use of hanging equipment to prevent carcasses contamination	Yes No	-ref- 2.68 (1.31–5.48)	0.01	-	
2	Slaughtering knives are sanitized before use	Yes No	-ref- 1.91 (0.73–5.01)	0.18	-	
3	Having temperature control for scalding water	Yes No	-ref- 4.84 (2.72–8.61)	<0.001	4.84 (2.72–8.61)	<0.001
4	Eviscerated carcasses	No Yes	-ref- 1.32 (1.04–1.69)	0.02	-	

*OR = odds ratio, -ref- = reference class.

**Table 3:** Factors associated with the *Staphylococcus aureus* non-compliance of meat samples based on the univariable and multivariable mixed-effects logistic regression.

	Factors	Categories	Univariable analysis		Multivariable analysis	
			Odds ratio (95% CI)	*P*-Value	Adjusted OR* (95% CI)	*P*-Value
1	Separating equipment for clean and unclean area	Yes No	-ref- 1.57 (0.63–2.43)	0.06	-	
2	Slaughtering knives are sanitized before use	Yes No	-ref- 1.51 (1.01–2.25)	0.04	-	
3	Cutting knives are sanitized before use	Yes No	-ref- 1.56 (1.05–2.36)	0.03	-	
4	Having temperature control for scalding water	Yes No	-ref- 2.68 (1.29–5.55)	0.008	2.68 (1.29–5.55)	0.008
5	Separating slaughtering line for clean and unclean area	Yes No	-ref- 2.48 (0.49–12.67)	0.15	-	

*OR = odds ratio, -ref- = reference class.

**Table 4:** Factors associated with the *Enterococcus* spp. non-compliance of meat samples based on the univariable and multivariable mixed-effects logistic regression.

	Factors	Categories	Univariable analysis		Multivariable analysis	
			Odds ratio (95% CI)	*P*-Value	Adjusted OR* (95% CI)	*P*-Value
1	Separating workers for clean and unclean area	Yes No	-ref- 1.58 (0.90–2.78)	0.11	-	
2	Use of hanging equipment to prevent carcasses contamination	Yes No	-ref- 2.10 (1.10–4.02)	0.03	-	
3	Having temperature control for scalding water	Yes No	-ref- 3.38 (2.01–5.69)	< 0.001	3.38 (2.01–5.69)	< 0.001
4	Eviscerated carcasses	No Yes	-ref- 1.39(1.09–1.79)	0.01	-	

*OR = odds ratio, -ref- = reference class.

**Table 5:** Factors associated with the coliforms non-compliance of meat samples based on the univariable and multivariable mixed-effects logistic regression.

	Factors	Categories	Univariable analysis		Multivariable analysis	
			Odds ratio (95% CI)	*P*-Value	Adjusted OR* (95% CI)	*P*-Value
1	Slaughterhouses have the system to treat water used during slaughtering process	Yes No	-ref- 1.73 (1.19–2.61)	0.01	-	
2	Separating workers for clean and unclean area	Yes No	-ref- 2.36 (1.11–5.05)	0.02	-	
3	Separating equipment for clean and unclean area	Yes No	-ref- 1.72 (1.26–2.36)	0.01	-	
4	Use of hanging equipment to prevent carcasses contamination	Yes No	-ref- 1.91 (0.75–4.85)	0.17	-	
5	Slaughtering knives are sanitized before use	Yes No	-ref- 1.98 (1.48–2.65)	0.01	-	
6	Having temperature control for scalding water	Yes No	-ref- 3.01 (1.47–6.15)	0.003	3.01 (1.47–6.15)	0.003
7	Eviscerated carcasses	No Yes	-ref- 1.89 (0.89–4.00)	0.09	-	

*OR = odds ratio, -ref- = reference class.

**Table 6:** Factors associated with the *Escherichia coli* non-compliance of meat samples based on the univariable and multivariable mixed-effects logistic regression.

	Factors	Categories	Univariable analysis		Multivariable analysis	
			Odds ratio (95% CI)	*P*-Value	Adjusted OR* (95% CI)	*P*-Value
1	Slaughterhouses have the system to treat water used during slaughtering process	Yes No	-ref- 1.76 (0.93–3.35)	0.08	-	
2	Separating workers for clean and unclean area	Yes No	-ref- 2.36 (1.32–4.22)	0.01	-	
3	Use of hanging equipment to prevent carcasses contamination	Yes No	-ref- 2.29 (1.21–4.35)	0.02	-	
4	Slaughtering knives are sanitized before use	Yes No	-ref- 1.67 (0.97–2.90)	0.06	-	
5	Cutting knives are sanitized before use	Yes No	-ref- 1.62 (0.93–2.82)	0.09	-	
6	Having temperature control for scalding water	Yes No	-ref- 2.58 (1.52–4.38)	<0.001	2.69 (1.58–4.56)	<0.001
7	Building system	Close Open or semi-close	-ref- 1.99 (1.20–3.29)	0.01	-	
8	Eviscerated carcasses	No Yes	-ref- 1.85 (1.07–3.20)	0.01	1.96 (1.14–3.38)	0.02

*OR = odds ratio, -ref- = reference class.

**Table 7:** Factors associated with the *Salmonella* spp. non-compliance of meat samples based on the univariable and multivariable mixed-effects logistic regression.

	Factors	Categories	Univariable analysis		Multivariable analysis	
			Odds ratio (95% CI)	*P*-Value	Adjusted OR* (95% CI)	*P*-Value
1	Slaughterhouses have the system to treat water used during slaughtering process	Yes No	-ref- 1.52 (0.94–2.48)	0.09	-	
2	Separating workers for clean and unclean area	Yes No	-ref- 1.59 (1.18–2.19)	0.02	-	
3	Separating equipment for clean and unclean area	Yes No	-ref- 1.74 (1.10–2.76)	0.01	1.65 (1.04–2.61)	0.02
4	Use of hanging equipment to prevent carcasses contamination	Yes No	-ref- 1.67 (1.03–2.69)	0.04	-	
5	Cutting knives are sanitized before use	Yes No	-ref- 1.23 (0.98–1.47)	0.08	-	
6	Building system	Close Open or semi-close	-ref- 1.84 (1.27–2.69)	0.001	1.79 (1.23–2.60)	0.002

*OR = odds ratio, -ref- = reference class.

## Discussion

This study provided nationwide data on bacterial contamination of chicken meat from all approved slaughterhouses in Thailand. The major findings were the high non-compliance for *Salmonella* spp. and *Escherichia coli* contamination in meat samples. Furthermore, this study showed that the non-compliance of meat samples due to bacterial contamination based on regulatory standard criteria was related to slaughterhouse structure, hygienic practices, and product characteristics.

The proportion of non-compliant status due to aerobic plate count (APC), *S*. *aureus*, *Enterococcus* spp., coliforms, *E*. *coli*, and *Salmonella* spp. determined in this study present a national baseline. The 95% CI of the proportion calculated herein also provides the lower and upper bound for statistical estimation that accounts for sampling variation offering a border of interpretation. Overall, compared to other reports in Thailand, our bacterial contamination results are in agreement with the previous study conducted at the provincial and regional levels that focused on meat contamination in slaughterhouses [34] but differed from other studies [20,35,36]. The variability may be a result of differences in study designs, year of the study conducted, and sampling variations.

In comparison with reports from other countries, diverse findings were found according to their safety limitations. For APC as a hygienic indicator [37], the proportion of non-compliant meat samples in this study was lower than the finding reported in Moroccan slaughterhouses (29.2%) but higher than those of Indian slaughterhouses (14%) [38,39]. Generally, *S*. *aureus* contamination usually reflects as a consequence of inadequate control measures and poor personal hygiene [6,16]. Studies conducted in Algeria and European countries revealed a higher *S*. *aureus* non-compliance ranging between 38.5 and 46.7% compared to the findings in this study [6,40]. Meat samples contaminated with *Enterococcus* spp. found in this study were consistent with those observed in Slovakia and Brazil which ranged from 1.8 to 50.9% [41,42]. Those might be indicated contamination from slaughtering equipment, wastewater, and slaughterhouse environment [43–45]. Our findings showed the proportion of coliforms and *E*. *coli* non-compliant meat samples to be lower than the 22% of coliforms and 43% of *E*. *coli* contamination recorded in a previous study [38]. These occurrences might result from unsatisfactory sanitary conditions of slaughtering as well as fecal contamination [46,47]. Given that the majority of *Salmonella* spp., a well-known foodborne pathogen, was slightly lower than the findings in previous studies in other countries, with isolation rates ranging from 36 to 56% [48–50]. On the contrary, other reports had indicated lower amounts of the *Salmonella*-positive samples at 7.1% and 9.5% in European countries and South Korea, respectively [6,14].

The present study also determined the factors associated with the non-compliance of the meat samples in the slaughterhouses. According to the multivariable mixed-effect logistic regression analysis, the results highlighted that the scalding process without scalding water temperature control or improper scalding had a significant impact on the non-compliant meat samples for APC, *S*. *aureus*, *Enterococcus* spp., coliforms, and *E*. *coli* contamination. Our findings were in agreement with other studies that revealed an increase in bacterial contamination on chicken carcasses following the scalding process due to the differences in the scalding temperature, the amount of freshwater added during the scalding process, and the immersion time [51,52]. The findings in this study are also supported by results from several studies [53–58] that indicate the role of proper scalding process in significantly reduced bacterial contamination. The scalding temperature had a great impact; in particular, when the scalding temperature reached 60°C, bacteria on chicken carcasses reduced approximately by 2 log CFU/cm^2^ [57]. In this study, based on livestock authorities’ inspection, the scalding water temperature ranged between 55–65°C. This finding clearly showed that temperature control during the scalding process significantly reduced bacterial contamination in chicken carcasses. The temperature observed in this study was in agreement with a previous report in Thailand that revealed the temperature of scalding water with the range of 62–66°C [59], while reports from other countries showed the temperature range between 50–70°C [51,52,56].

In addition to the improper scalding, product characteristic was associated with *E*. *coli* non-compliant meat samples. Our result indicated that eviscerated carcasses were more likely than non-eviscerated carcasses to be contaminated with *E*. *coli*. This finding was in concordance with other studies showing similar results, which found a significant increase in *E*. *coli* after evisceration process [60]. It is probable that bacterial cross-contamination from leaking intestinal content, giblets, and equipment to carcasses occurred during the evisceration process [61]. Similar observations were noted for increasing the level of *E*. *coli* on chicken carcasses during the evisceration process [16,54,60]. Given that more than 60% of chicken carcasses processed in slaughterhouses were eviscerated in this study, it is important to raise slaughterhouse workers’ awareness of *E*. *coli* contamination.

*Salmonella* spp. could be found as early as the chicken farms, transport vehicles, live bird crates, and equipment along the slaughtering area [14,15,62]. In this study, the cross-contamination from equipment in slaughtering lines was identified as the factor associated with the *Salmonella* spp. contamination in meat. This finding was in a line with results from other studies demonstrating slaughter equipment including tables, gloves, baskets as well as the slaughterhouse environment as the sources of cross-contamination for carcasses [15,52,63–66]. Additionally, we found an association between open or semi-closed slaughterhouses and a higher risk of *Salmonella* spp. contamination in chicken meat. In contrast to closed slaughterhouses, these facilities allow for more unfettered contact between flies and carcasses, thereby increasing the possibility of cross-contamination since some flies presented in slaughterhouses (approximately 15%) could carry viable *Salmonella* spp. [67].

To the best of our knowledge, this was the first study in Thailand to identify the risk factors associated with non-compliant meat samples owing to bacterial contamination in slaughterhouses through the official nationwide survey conducted by the livestock authorities. Based on the findings from the present study, we recommend that the authorities should supervise slaughterhouse personnel to enhance the slaughtering processes and facilities including proper scalding, evisceration, slaughtering equipment, and slaughterhouse structure. Also, implementing training or education programs for stakeholders or slaughterhouse staff for safe meat production would be prudent. Moreover, the public should be made well-aware of the high prevalence of foodborne pathogens such as *Salmonella* spp. on the meat. This study offers information on bacterial contamination and associated risk factors for the bacterial contaminations based on nationwide data, and thereby contributes to the national need for information on this subject. The data provided in this study could be used as a basic information that can be gathered for future use by international organizations. We hope that our results could be also useful to other chicken slaughterhouses with similar conditions outside Thailand.

## Conclusions

In spite of dedicated efforts from the government authorities to implement control over bacterial contamination in slaughterhouses, especially by foodborne pathogens such as *Salmonella* spp., the challenge to achieve compliance to the regulations persists. In this study, we identified several risk factors for bacterial contamination of chicken meat from slaughterhouses across Thailand. Our results indicated that the scalding process, evisceration, equipment, and slaughterhouse structure were the critical issues that warrant improvement. Therefore, a specific strategic plan based on these issues needs to be formulated.

## Supporting information

S1 Table Department of Livestock Development microbiological criteria and laboratory method for livestock products. (DOCX)

S2 Table Factors used for the mixed-effects logistic regression analysis. (DOCX)
