# Supplementary material for: Bacterial contamination of chicken meat in slaughterhouses and the associated risk factors: A nationwide study in Thailand
Source: PLoS One. 2022 Jun 8;17(6):e0269416. doi: 10.1371/journal.pone.0269416 (PMC9176793; doi:10.1371/journal.pone.0269416)
Supplement: S1 Table — (DOCX) [file pone.0269416.s001.docx]

**S1 Table. Department of Livestock Development Microbiological Criteria and Laboratory Method for Livestock Products.**

| **Item** | **Microorganism** | **Criteria*** | **Method** |
| --- | --- | --- | --- |
| 1 | Aerobic plate count | ≤5 × 10^5^ (CFU/g) | FDA BAM *Online*, 2001 (Chapter 3) |
| 2 | *Staphylococcus aureus* | ≤100 (CFU/g) | ISO 6888-1: 1999 |
| 3 | *Enterococcus* spp. | ≤1,000 (CFU/g) | Nordic Committee on Food Analysis, No. 68 5^th^ Edition (2011) |
| 4 | Coliforms | ≤5,000 (CFU/g) | FDA BAM *Online*, 2013 (Chapter 4) |
| 5 | *Escherichia coli* | ≤100 (CFU/g) | FDA BAM *Online*, 2013 (Chapter 4) |
| 6 | *Salmonella* spp. | Not detected in 25 g | ISO 6579-1: 2017 |

***** Microbiological Criteria of the Department of Livestock Development
