# Supplementary material for: Bacterial contamination of chicken meat in slaughterhouses and the associated risk factors: A nationwide study in Thailand
Source: PLoS One. 2022 Jun 8;17(6):e0269416. doi: 10.1371/journal.pone.0269416 (PMC9176793; doi:10.1371/journal.pone.0269416)
Supplement: S2 Table — (DOCX) [file pone.0269416.s002.docx]

**S2 Table. Factors used for the mixed-effects logistic regression analysis.**

| **Factors** | **Categories** |
| --- | --- |
| **Slaughterhouse management practices** | |
| Water Source used in slaughterhouses | 1 = tap water, 0 = other sources (surface water, groundwater, etc.) |
| Slaughterhouses have the system to treat water used during  slaughtering process | 1 = yes, 0 = no |
| Separating workers for clean and unclean area | 1 = yes, 0 = no |
| Separating equipment for clean and unclean area | 1 = yes, 0 = no |
| Hand cleaning practice before entering the slaughtering area | 1 = yes, 0 = no |
| Changing protective clothes and boots before entering the slaughtering area | 1 = yes, 0 = no |
| Use of hanging equipment to prevent carcasses contamination | 1 = yes, 0 = no |
| Slaughtering knives are sanitized before use | 1 = yes, 0 = no |
| Cutting knives are sanitized before use | 1 = yes, 0 = no |
| Having temperature control for scalding water | 1 = yes (temperature between 55-65°C), 0 = no |
| **Slaughterhouse designated and facilities** | |
| Building system | 1 = close system, 0 = open or semi-close system |
| Separating slaughtering line for clean and unclean area | 1 = yes, 0 = no |
| **Product characteristics** | |
| Eviscerated carcasses | 1 = yes, 0 = no (un-eviscerated carcasses) |
